# Rhabdomyolysis and Acute Renal Impairment in a Patient with Hypothyroidism: A Case Report

**DOI:** 10.1155/2014/139170

**Published:** 2014-04-13

**Authors:** Arshi Naz, Mayada Issa

**Affiliations:** ^1^Department of Neurology, West Virginia University, 1 Medical Center Drive, Morgantown, WV 26505, USA; ^2^Department of Medicine, West Virginia University, 1 Medical Center Drive, Morgantown, WV 26505, USA

## Abstract

We report the case of a 33-year-old male with hypothyroidism who developed acute renal impairment with rhabdomyolysis after strenuous physical activity (snow shoveling). His thyroid function test confirmed marked hypothyroidism. Severe elevation of serum CK consistent with rhabdomyolysis was noted and an elevated creatinine indicated acute renal impairment. Patient's condition improved significantly after starting him on thyroid hormone replacement therapy and aggressive hydration. Acute renal impairment with rhabdomyolysis in patients with hypothyroidism is quite rare and we expect that this case report adds to the existing literature on this subject. We also emphasize that thyroid status should be evaluated in patients with unexplained acute renal impairment and presenting with the symptoms of muscle involvement.

## 1. Introduction

The effects of hypothyroidism on the renal function are fairly well established. These effects include histological changes as well as physiological changes. Most notable physiological changes include decrease in renal blood and plasma flow, decrease in glomerular filtration rate (GFR), alterations in water and electrolyte balance especially hyponatremia, and changes in tubular secretory and absorptive capacity [1]. The changes to renal functions are subtle in most patients with hypothyroidism and are often overlooked in clinical setting. Moreover, this association is often inferred when patient's renal impairment is improved by thyroid hormone replacement therapy.

Muscle disorders and other muscular manifestations are also common with hypothyroidism. These muscular manifestations may range from mere stiffness, weakness, or elevated creatinine phosphokinase to more serious manifestations such as Hoffman's syndrome or rhabdomyolysis [2, 3]. Histological changes in muscles may be observed in hypothyroidism as well. Although the association between muscle disorders and hypothyroidism is well noted, the association of rhabdomyolysis characterized by muscle necrosis and myoglobinuria with hypothyroidism is very rare.

We report the case of a 33-year-old male with hypothyroidism who developed acute renal impairment with rhabdomyolysis. This case report joins the list of the selected few case reports that reported hypothyroid acute renal impairment along with the association of rhabdomyolysis.

## 2. Case Report

A 33-year-old Caucasian male was transferred to our hospital from outside facility with acute renal impairment. The patient had no significant past medical history other than gastroesophageal reflux disease (GERD) and he was not known to be on any medication. He has history of tobacco use (rubbing and snuffing) for 20 years and drinks 3 cans of beer per week.

Patient presented with the chief complaint of bilateral lower extremities edema and generalized muscle weakness and cramps. These symptoms were noticed two days prior to the admission. Two months prior to the admission, the patient had also complained of tiredness and constipation. He had not been sleeping well at night. He denied being in unusual stress situation. He was also feeling colder than other people around him. Prior to the admission, the patient noted a weight gain of 10 pounds in 2 days. Patient denied any history of seizure, trauma, or fall recently although he mentioned that he had been doing a lot of snow shoveling over the past two weeks before admission.

On presentation, the patient had a body temperature of 36.6°C, pulse of 78 per minute, respiratory rate of 20, and blood pressure of 193/115 mm Hg. The patient was obese with a body mass index of 38.1 Kg/m^2^. On physical examination, he was alert and oriented. His conjunctivas were clear and pupils were equal, round, and reactive to light and accommodation. An enlarged, rubbery thyroid was palpable. Chest was clear on auscultation bilaterally and S1/S2 was normal with no murmur. Abdomen was soft and nontender. Pitting edema was noted in lower extremities bilaterally with slight muscle tenderness. No evidence of dehydration was found. No lymphadenopathy was noted. No motor or sensory disturbances were noted.

Blood tests showed a creatinine of 1.54 (reference: 0.62–1.27 mg/dL). His GFR was 52 (reference: >59 mL/min/1.73 m^2^). Blood urea nitrogen (BUN) was 13 (reference: 8–26 mg/dL). His CK was 7200 (reference: 48–222 U/L). Sodium was 141 (reference: 136–145 mmol/L), potassium was 4.2 (reference: 3.5–5.1 mmol/L), and chloride was 99 (reference: 96–111 mmol/L). Urine electrolytes were also measured. Fractional excretion of sodium (FE_Na_) was 0.88 pointing towards prerenal cause of renal impairment.

His TSH was 145.7 (reference: 0.3–5.9 *μ*lU/mL). TSH with free T4 reflex was 117.13 (reference: 0.3–5.9 *μ*IU/mL) and thyroxine free (free T4) was <0.3 (reference: 0.6–1.1 ng/dL). Thyroid peroxidase (TPO) antibody was 150 (reference: <40 IU/mL).

Liver function tests showed an aspartate aminotransferase (AST) of 148 (reference: 8–48 U/L), alanine transaminase (ALT) of 82 (reference: 7–55 U/L), and lactate dehydrogenase (LDH) of 239 (reference: 98–192 U/L). Lipid panel showed cholesterol of 365 (reference: <200 mg/dL), high-density lipoprotein (HDL) of 42 (reference: >39 mg/dL), and triglyceride (TG) of 257 (reference: <150 mg/dL).

White blood cell (WBC) count was 6.9 (reference: 3.5–11 10^3^/*μ*L). Erythrocyte sedimentation rate (ESR) was 12 (reference: 0–15 mm/hr). C-reactive protein (CRP) was 0.27 (reference: <0.8 mg/dL). B-type natriuretic peptide (BNP) was 20 (reference: <100 pg/mL). Hemoglobin (HGB) was 12.3 (reference: 13.1–17.3 g/dL). Ferritin was 515 (reference: 24–336 ng/mL). Serum iron was 93 (reference: 50–160 *μ*g/dL). Total iron binding capacity (TIBC) was 333 (reference: 260–400 *μ*g/dL). Iron saturation was 28 (reference: 20–50%). Urine analysis was negative for hematuria and proteinuria.

Ultrasound of thyroid showed a small thyroid that was hypoechoic and heterogeneous with diminished vascularity. These findings, although nonspecific, are suggestive of prior episodes of thyroiditis or long standing hypothyroidism. Ultrasound of kidney was unremarkable. Transthoracic echocardiogram (TTE) was within normal limit.

The patient was started on levothyroxine at 125 mcg and aggressive hydration. The patient was initially given IV fluids at a rate of 200 mL/hr for 8 hours in addition to the oral hydration. The fluids were subsequently tapered off. His creatinine decreased to 1.44 mg/dL and his CK decreased to 5409 U/L after a day of treatment. Leg edema and muscle cramps started improving rapidly after a day of treatment. At the time of presentation, the patient was also given IV hydralazine 10 mg to lower his blood pressure. He was subsequently continued on hydralazine 25 mg every six hours to manage his hypertension. Patient's condition continuously showed improving trend over a 2-day period after which he was discharged.

On follow-up after four months, the patient's creatinine decreased to 0.9 mg/dL and his GFR was >60 (reference: >59 mL/min/1.73 m^2^). His TSH was 0.469 (reference: 0.3–5.9 *μ*lU/mL). TSH with free T4 reflex was 6.22 (reference: 0.3–5.9 *μ*IU/mL). His HGB was 15.3 (reference: 13.1–17.3 g/dL). His AST level was 47 (reference: 8–48 U/L).

## 3. Discussion

Kidney and thyroid functions are closely interrelated and their interactions have been fairly well established [4–9]. Thyroid hormones are not only necessary for the growth and development of kidney; they also play a significant role in renal physiology. Hypothyroidism can affect renal hemodynamics and can also induce water and sodium retention leading to hyponatremia. The pathophysiology of renal dysfunction in hypothyroidism is still unclear. Renal impairment with hypothyroidism is thought to be due to reduced cardiac output and increased systemic and renal vasoconstriction leading to reduced renal blood and plasma flow and decreased GFR. Hemodynamic alterations in hypothyroidism, although important, are often subtle and cannot always fully explain the observed renal dysfunction. A study by Den Hollander et al. [10] noted that variations in the blood pressure during thyroid dysfunction are too small to explain the observed changes in renal function. Apart from the effect of hypothyroidism on cardiac output, triiodothyronine (T3) has a direct effect on systemic vascular resistance which can influence renal blood flow directly thus causing renal dysfunction [10, 11]. Moreover, brain natriuretic peptide (BNP) levels correlate with free T3 and T4 levels [11], which may affect cardiac output and GFR. Another mechanism that can explain renal dysfunction in hypothyroidism is the influence that thyroid hormones have on tubular secretion of creatinine. Thyroxine (T4) is known to regulate Na^+^/Ca^2+^ channels and Na^+^/K^+^-ATPase activity in the sarcoplasmic reticulum of nephrons [12]. Another important mechanism of renal dysfunction in hypothyroidism is through muscle involvement. Muscle disorders and other muscular manifestations are common with hypothyroidism as creatinine production in muscles is dependent on thyroid status. These muscular manifestations may range from mere stiffness, weakness, or elevated creatinine phosphokinase to more serious manifestations such as Hoffman's syndrome or rhabdomyolysis [3]. Acute renal impairment due to rhabdomyolysis induced by hypothyroid state is quite rare and only a handful of case reports are available [13–20]. This case report adds to this list and presents researchers with adequate data to characterize the rare occurrence of acute renal impairment due to hypothyroidism induced rhabdomyolysis.

Rhabdomyolysis is associated with a wide variety of causes such as seizures, trauma, alcohol and drug abuse, strenuous exercise, drug induced and others. Rhabdomyolysis occurs frequently, but it is usually asymptomatic with abnormalities limited to lab findings. In severe cases, rhabdomyolysis can lead to electrolyte disorders and acute renal failure. There are many factors that can contribute to the development of more severe clinical symptoms in rhabdomyolysis such as hypovolemia, hyperthermia, electrolyte disorders, congenital muscle disorders, and hypothyroidism. As noted before, hypothyroidism is a quite uncommon underlying factor behind rhabdomyolysis that can not only trigger its onset but also affect its severity. In our case, patient's rhabdomyolysis was triggered by exertion (snow shoveling) and the underlying hypothyroid status exacerbated its severity leading to acute renal impairment. Rhabdomyolysis was evident in our case based on severe elevation of serum CK to 7200 (reference: 48–222 U/L). Although there is no well-established serum CK level at which rhabdomyolysis is pronounced to occur, a concentration of five times the upper limit of the normal value has been suggested [21]. Serum CK levels are also considered to be a predictive measure indicating development of acute renal failure [21]; however, serum CK levels do not correlate with the severity of renal failure [22]. A creatinine of 1.54 (reference: 0.62–1.27 mg/dL) was noted indicating acute renal impairment. In each of the case reports that reported acute renal failure due to hypothyroidism induced rhabdomyolysis [13–20], improvements were seen in patients after starting thyroid hormone replacement therapy. Cai and Tang [20] reported two patients who also required blood purification. Our patient improved significantly after starting him on thyroid hormone replacement therapy and aggressive hydration.

Our patient had edema in lower extremities bilaterally with slight muscle tenderness. Edema has been associated with thyroid dysfunction as studies suggest that thyroid hormones affect small vessel permeability leading to leakage of albumin from the vessels [23–25]. Decrease in renal plasma flow, GFR, and free water clearance associated with hypothyroidism can also contribute to edema in patients [26]. Hyponatremia is seen in patients with hypothyroidism [27], but it was not noted in our patient based on normal serum sodium level thus eliminating hyponatremia as the cause of rhabdomyolysis.

Hypothyroidism can adversely affect the hematological system and lead to development of anemia. In a study designed to measure the prevalence of anemia in patients with overt and subclinical hypothyroidism, Mehmet et al. [28] found that the frequency of anemia in patients with overt and subclinical hypothyroidism is statistically more significant than in the healthy control group. They also noted that anemia of chronic disease is the most frequently seen anemia type in patients with overt and subclinical hypothyroidism. We note that the patient reported here had anemia of chronic disease with elevated Ferritin and normal TIBC with HGB of 12.3. After treatment with levothyroxine, patient's HGB returned to normal when followed up four months after hospital discharge.

Our patient had an elevated level of TPO antibody indicating chronic autoimmune thyroiditis as the cause of his hypothyroidism. Chronic autoimmune thyroiditis has been cited as the most frequent cause of hypothyroidism [29].

## 4. Conclusions

In this case report, we report an occurrence of acute renal impairment due to hypothyroidism induced rhabdomyolysis. Acute renal impairment with rhabdomyolysis in patients with hypothyroidism is quite rare and we expect that this case report adds to the existing literature on this subject and provides researchers with adequate data to characterize this disorder further. We also emphasize that thyroid status should be evaluated in patients with unexplained acute renal impairment and presenting with the symptoms of muscle involvement such as myalgia and generalized weakness with minimal exertion.
